# Assessment of sexual dysfunction and associated factors among patients with schizophrenia in Ethiopia, 2017

**DOI:** 10.1186/s12888-018-1738-3

**Published:** 2018-05-29

**Authors:** Tolesa Fanta, Kibrom Haile, Dessie Abebaw, Dawit Assefa, Getahun Hibdye

**Affiliations:** 1 Amanuel Mental Specialized Hospital Research and Training Department, Addis Ababa, Ethiopia; 2 Amanuel Mental Specialized Hospital, Addis Ababa, Ethiopia; 3 Amanuel Mental Specialized Hospital Clinical Service Department, Addis Ababa, Ethiopia

**Keywords:** Sexual dysfunction, Schizophrenia, Amanuel hospital

## Abstract

**Background:**

Sexual dysfunction is remarkably prevalent amongst psychiatric patients than general population. This might be due to either the nature of the illness itself or the unwanted effect of the medication they are taking for the illness which limits the capability of forming interpersonal and sexual relationships. This issue is rarely raised in developing countries, and the aim of this study was to assess magnitude and factors contributing to sexual dysfunction among patients with Schizophrenia.

**Method:**

Hospital based cross sectional study was conducted at Amanuel Mental Specialized Hospital from January to June 2017. The sample required for this study was determined by using single population proportion formula and the final sample size was 423; and systematic random sampling was used to select participants. We used Change in Sexual Functioning Questionnaire to measure sexual dysfunction. The collected data was cleaned, interred in to Epi data and transferred to SPSS version 20 for farther analysis. The OR with 95% CI was used to measure association and *P*-value < 0.05 was used as statistically significant.

**Result:**

A total of 422 patients with Schizophrenia were involved in the study. The prevalence of General Sexual dysfunction was 82.7%; and in male and female patients the prevalence was 84.5 and 78.6% respectively. Marital status (Unmarried, Divorced and widowed, history of relapse and poor quality of life were associated significantly to global sexual dysfunction.

**Conclusion:**

The magnitude of Sexual dysfunction was found to be high among patients with schizophrenia and it is associated with different factors like unmarried, divorced, widowed, relapse and poor quality of life. Treating physicians should be conscious to sexual dysfunction during evaluation and treatment of patients with Schizophrenia. Special attention should be given to single, divorced, widowed patients and patients with history of relapse to improve quality of life of this patients.

## Background

Sexual life is a natural and complex component of human behaviors that is determined by many physiological and psychological factors. Sexual dysfunction is a public health issue which affects an estimate of 43% women and 31% men in US [1]. The commonest dysfunction among women is sexual desire dysfunction complained by around 30 % of women. The commonest dysfunctions among men are erectile dysfunction and premature ejaculation. Any problem in main area of sexual behavior; interest, arousal, orgasm/ejaculation and like can arise as the result of either pathophysiological or psychological mechanisms [2].

Sexual dysfunction is extremely prevailing in psychiatric patients than general population. This is related to either the nature of the illness itself (negative symptoms like avolition, anhedonia and blunted affect) or the unwanted effect of the medication they are taking for the illness (effect on prolactin secretion and obesity) which limits the capability of forming interpersonal and sexual relationships [1].

The peak age of onset of schizophrenia in both sexes is during the reproductive period. Consequently impaired sexual functioning among persons with schizophrenia can affect their ability to have a family, and, thus, to fulfill traditional social expectations [3]. The role of antipsychotic drugs in sexual functioning of people with schizophrenia is becoming a recent concern of researchers since this side-effect may decrease adherence to treatment, especially among males, because they are more concerned about sexual functioning than females) [4].

Sexual dysfunction has many impacts on patients with Schizophrenia. It has direct or indirect association with quality of life, adherence, difficulty to form and maintain family, and lastly may develop depression and suicidality [3, 5, 6].

Despite the importance and high prevalence of the problem, this patients do not inform the problem either due to feeling of discomfiture or for the reason that they do not view it as a treatable problem [6]. In other hand psychiatrists and other specialists significantly undervalue or even neglect the existence of the problem probably due to embarrassment of talking about sexual problems with patients, lack of time and viewing difficulties in this area as minor compared to psychotic symptoms. In spite of these realities there are limited or no researches conducted in this country regarding sexual dysfunction and its influence on patients with Schizophrenia. Therefore, this study aims to evaluate the prevalence of sexual dysfunction among patients with schizophrenia and see if there is any association between socio-demographic factors, different clinical factors like medication related factors, co-morbid physical or psychiatric conditions, and substance use and sexual dysfunction among patients with Schizophrenia.

## Methods

### Study design and study period

Institutional based cross sectional study was conducted from January to June 2017.

### Study area

The study was conducted at Amanuel Mental Specialized Hospital (ASMH) located in the country’s capital, Addis Ababa. Amanuel mental specialized hospital is the only mental specialized hospital where patients mainly afflicted with severe mental illness, including schizophrenia, are treated. The hospital gives service for patients from all over the country. It has a case load of more than 10,000 patients per month and schizophrenia is the number one diagnosis, diagnosed in more than 60% of the patients visiting the hospital.

### Population

All patients with Schizophrenia who are on follow up at AMSH are the source populations and people with Schizophrenia in the age group 18 & above who were on treatment at AMSH during the study period were study population.

### Eligibility criteria

All patients with Schizophrenia in age group 18 and above were included in the study and the patients in exacerbation phase were excluded from the study.

### Sample size

The minimum number of sample required for this study wasdetermined by using single population proportion formula and the final sample size for this study with 10% nonresponse rate was 423.

### Sampling procedure

Systematic random sampling technique with interval of 11 was used to select the participants from 4885 patients with schizophrenia came for follow up during data collection period.

### Study variables

The outcome variable for this study was Sexual Dysfunction. Socio-demographic factors, duration of the illness, duration on treatment, medication, dosage and frequency, comorbid known chronic medical illness, history of admission and relapse, adherence to drug, Quality of life, Suicide, Depression and history of substance use were explanatory variables for this study.

### Instruments

The gold standard instrument which is Structured Clinical Interview for DSM- ΙѴ-TR axis Ι disorders (SCID) was used to confirm a diagnosis of Schizophrenia. Sexual dysfunction was measured by using Changes in Sexual Functioning Questionnaires (CSFQ-14). It has separate forms for female (CSFQ –F-C) and for Male (CSFQ-M-C) Clinical Version. It contains14 items and is used to assess the presence/absence of sexual dysfunction in study participants. All the 14 items should be answered on a five Likert scale to assess global sexual dysfunction. The score < =47 for male and < =41 for female indicates the presence of global sexual dysfunction. The tool can also measure the sexual dysfunction components: Pleasure (Item 1), Desire/frequency (Item 2 and 3), Desire/interest (Item 4, 5 and 6), Arousal/erection (Item 7, 8 and 9) and Orgasm/ejaculation (Item 11, 12 and 13) It has Cronbach’s α of 0.91 and 0.93 for male and female scales, respectively [7, 8]. WHOQOL-BREF was used to measure quality of life. This instrument is cross culturally validated and currently in use in different languages [9]. PHQ-9 was used to measure depression in patients with schizophrenia. This instrument has sensitivity of 86% and specificity of 67%in diagnosing depression [10]. Eight –item Morisky medication adherence Scale was used to measure medication adherence. It is valid and reliable with Cronbach’s α of 83%; and, sensitivity and specificity of 93 and 53% respectively [11]. The English version of the instruments was translated to local language and back retranslated to English by language professionals and psychiatrists.

### Data quality control

17 masters level mental health students were hired for data collection and two masters level mental health professionals were hired to supervise the data collectors. The data collectors were given a two days training on questionnaire and way of assessment. Pre-test was conducted 15 days before the start of actual data collection to know the time needed to complete one questionnaire and to know whether the questionnaire used is understandable to the study participants or not. The data collected during the pre-test was not included in the final analysis.

### Data processing and analysis

Data was coded and entered to Epi data and transferred to Statistical Package for Social Sciences version 20 (SPSS-20) for further analysis. Descriptive statistical analysis was used to estimate the frequencies and percentages of the variables. Bivariate and multivariate logistic regression analysis was used to see the association between outcome and explanatory variables. The strength of the association was measured by odds ratio with 95% CI and *P*-value less than 0.05 was considered as statistically significant.

### Ethical consideration

Ethical clearance was obtained from Amanuel Mental Specialized Hospital Ethical Review Committee. The Four Item Abbreviated Mental Test (AMT4) was used to measure the capacity of the patient to give consent. Then the purpose, importance and confidentiality of the information gathered was explained to each of the competent participant before the start of interview. Participants were also informed that they will never get any benefit because of participation in the study and no harm on them if they would not agree to participate or withdraw from participation during the data collection process. Finally, their willingness to be involved in the study was asked and written consent was obtained.

At the time of data collection the investigator, supervisor and data collectors followed ‘code of ethics’ and obeyed the rules & regulations of the hospital. Participant’s privacy was kept strictly at the time of data collection.

## Result

**Fig. 1:**
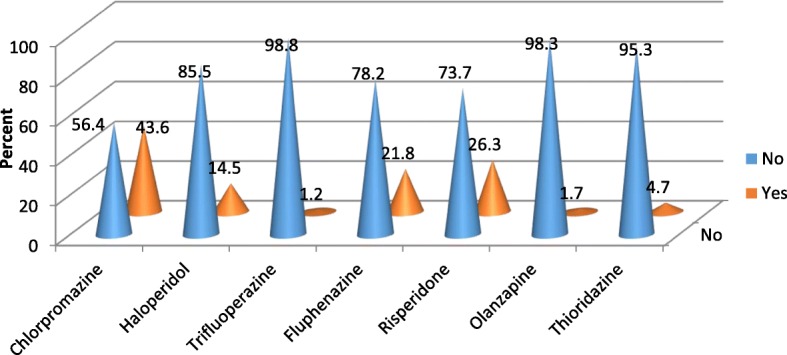
Antipsychotic Medications prescribed for the participants

**Fig. 2:**
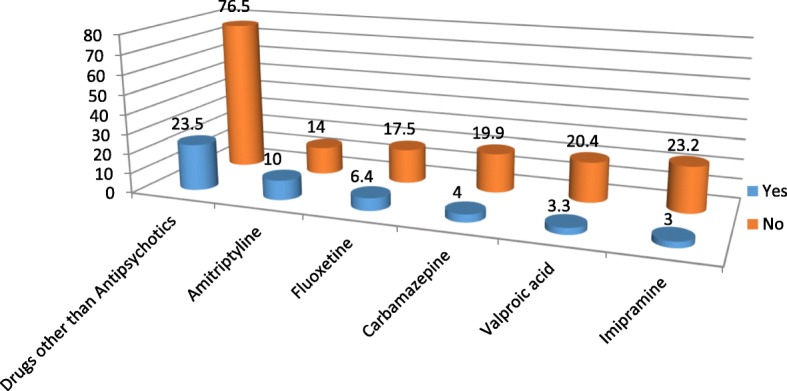
Medications prescribed for comorbid psychiatric problems

**Fig. 3:**
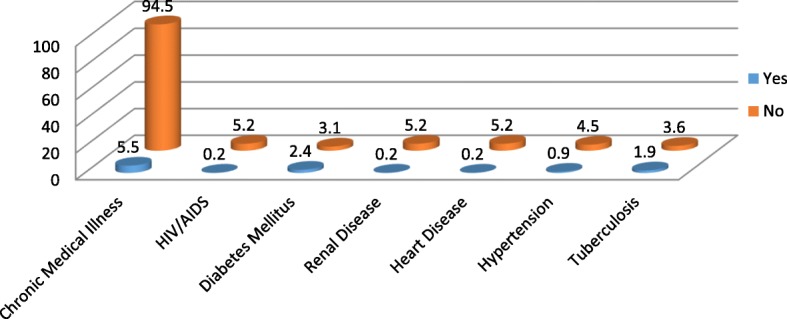
Magnitude of Chronic Medical Illness among Patients with Schizophrenia

**Fig. 4:**
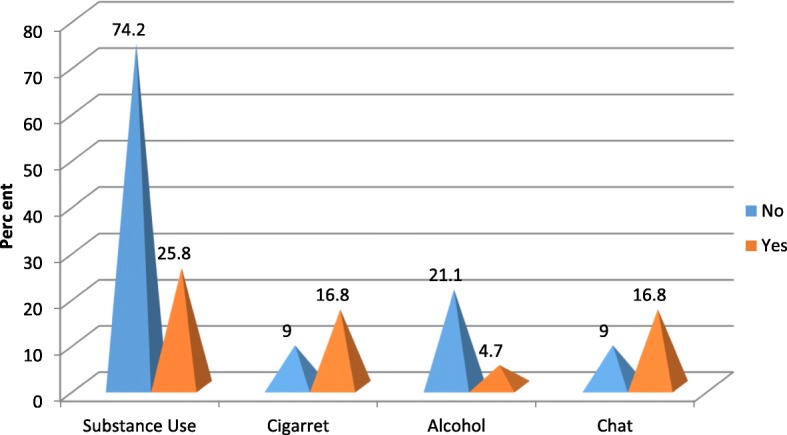
Magnitude of Substance Use among Patients with Schizophrenia

A total of 422 patients with schizophrenia participated on the study with response rate of 99.76%. Among the participants 290(68.7%) were male and 132(31.3%) were female in gender. The mean age of the participants is 35.46 with ± 9.25 standard deviation. Majority of the participants 353(83.6%) were from urban area. The most frequently prescribed antipsychotic drug is chlorpromazine 184(43.6%) followed by Resperidone 111 (26.3%) (Fig. 1). Amitriptyline is the most frequently prescribed drug among the medications ordered for other comorbid psychiatric conditions 42(41.6%) followed by Fluoxetine 27(26.7%) (Fig. 2). Most of the participants take their medications once in twenty four hours (Table 1). Among the study participants, 23(5.5%) were found to have at least one comorbid other medical illness. The most frequently occurring chronic medical illness in patients with Schizophrenia is Diabetes Mellitus 10(2.4%) followed by Tuber Closes (TB) 8(1.9%) (Fig. 3). Among the substance users 71(16.8%) use cigarette and khat users were 71(16.8%) (Khat and Cigarette are equally consumed) (Fig. 4). The median score of duration of the illness is 7 years with inter quartile range of 6, and the median score of duration on treatment is 6 years with inter quartile range of 10. The median score for frequency of admission is 1 with inter quartile range of 1, and the median score for frequency of relapse is 2 with inter quartile range of 2. The mean score of Quality of Life of the participants is 60.59 with standard deviation of ± 9.43 (Table 1).

**Table 1:** Distribution of participants by socio-demographic factors and clinical factors

No.	Variables	Variables category	Frequency (422)	Percentage (100%)
1	Age	18–24	35	8.3
		25–34	174	41.2
		35–44	150	35.5
		> = 45	63	14.9
2	Sex	Female	132	31.3
		Male	290	68.7
3	Marital Status	Married	154	36.5
		Single	224	53.1
		Divorced and Widowed	44	10.4
4	Ethnicity	Oromo	140	33.2
		Amhara	126	29.9
		Gurage	98	23.2
		Others*	58	13.7
5	Religion	Orthodox	228	54
		Protestant	79	18.7
		Muslim	115	27.3
6	Educational Status	No formal education	31	7.3
		Primary school	137	32.5
		High School	164	38.9
		Diploma	46	10.9
		Degree and above	44	10.4
7	Occupation	Private	135	32
		Governmental	42	11.1
		Unemployed	142	33.6
		Others(House wife, Daily labourers)	98	23.2
8	Residence	Urban	353	83.6
		Rural	69	16.4
9	Frequency of Chlorpromazine per day	Once/day	149	81.0
		> = 2	35	19
10	Frequency of Haloperidol per day	Once/day	46	75.4
		> = 2	15	24.6
11	Frequency of Trifluoperazine per day	Once/day	3	60
		> = 2/day	2	40
12	Frequency of Fluphenazineper day	Once/month	91	98.9
		> = 2/month	1	1.1
13	Frequency of Resperidone per day	Once/day	71	64.5
		> = 2/day	39	35.5
14	Frequency of Olanzapine per day	Once/day	5	62.5
		> = 2/day	2	37.5
15	Frequency of Thioridazine per day	Once/day	20	100
		> = 2/day	0	0
16	Duration of the illness	<=5 years	174	41.2
		6-10 years	119	28.2
		> = 11 years	129	30.6
17	Duration on treatment	<=5 years	207	49.1
		6-10 years	103	24.4
		> = 11 years	112	26.5
18	Admission	No	254	60.2
		Yes	168	39.8
19	Number of admission	<=1	96	57.1
		> = 2	72	42.9
20	Relapse	No	226	53.6
		Yes	196	46.4
21	Number of relapse	<=1	96	49
		> = 2	100	51
22	Depression	No	346	82
		Yes	76	18
23	Non-Adherence	No	203	48.1
		Yes	219	51.9
24	Poor Quality of life	No	217	51.4
		Yes	205	48.6
		Yes	0	0
25	Suicidal Ideation	No	373	88.4
		Yes	49	11.6
26	Suicidal Attempt	No	406	96.2
		Yes	16	3.8

### Prevalence of sexual dysfunction among patients with schizophrenia

**Fig. 5:**
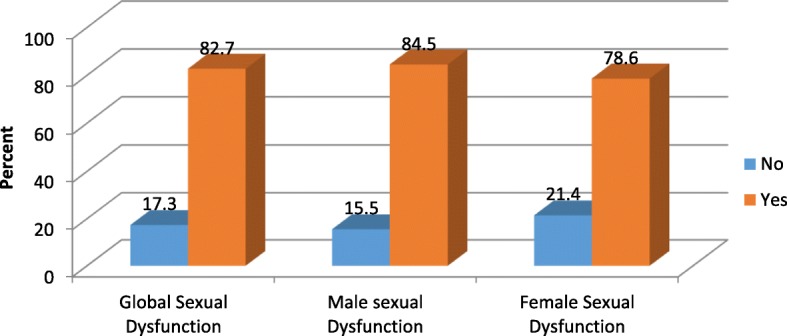
Global sexual Dysfunction and sexual Dysfunction across Male and Female participants

**Fig. 6:**
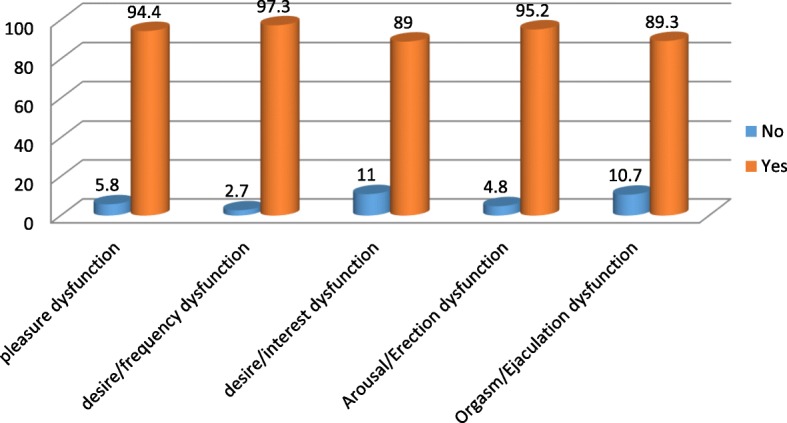
Components of Sexual Dysfunction among male participants

**Fig. 7:**
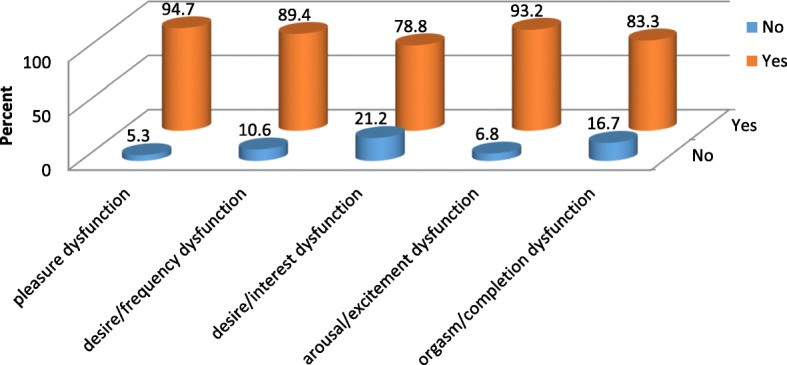
Components of Sexual Dysfunction among Female Participants

The prevalence of General sexual dysfunction among the study participants was 349(82.7%) with 95% confidence interval of (78.9, 86.3). The overall sexual dysfunction among male participants was 246(84.5%) with 95% confidence interval of (80.3, 88.7) and it was 103(78.6%) with 95% confidence interval of (71, 84.7) in female Schizophrenic patients (Fig. 5). Erectile dysfunction 277(95.2%) is highly prevalent followed by pleasure dysfunction 274(94.2%) in male participants (Fig. 6). In female participants who had sexual dysfunction the most prevalent sexual dysfunction was pleasure dysfunction 125(94.7%) followed by arousal/excitement dysfunction 123(93.2%) (Fig. 7).

### Bivariate and multivariate analysis

After bivariate logistic regression analysis, five variables (Marital status, Resperidone use, Relapse, Depression and Quality of life) met the requirement to proceed to multivariate logistic regression analysis. After multivariate analysis, marital status, history of relapse, and quality of life were found to be significantly associated with global sexual dysfunction. Compared to the married ones, being single, [aOR 4.19, 95% CI (2.30, 7.64)], and being divorced [aOR 2.86, 95% CI (1.03, 7.90)] were significantly associated with General sexual dysfunction. History of relapse, [aOR 2.21, 95% CI (1.25, 3.91)], and poor quality of life, [aOR 5.57, 95% CI (2.79, 11.09)] were also significantly associated with General sexual dysfunction at *p*-value< 0.05 (Table 2).

**Table 2:** Factors Associated With Sexual Dysfunction Among Patients With Schizophrenia At Amanuel Mental Specialized Hospital/2017

Explanatory variables	Variables category	Sexual Dysfunction		Bivariate and Multivariate Analysis		*P*-Value
		Absent	Present	Bivariate Analysis	Multivariate Analysis	
				COR (95% CI)	aOR (95% CI)	
Marital Status	Married	44	110	1:00	1:00	
	Single	23	201	3.49(2.01,6.10)	4.19(2.30,7.64)	0.000
	Divorced and Widowed	6	38	2.53(1.00,6.6.42)	2.86(1.03,7.90)	0.043
Resperidone	No	62	249	1.00	1.00	
	Yes	11	100	2.264(1.15,4.48)	1.69(0.82,3.51)	
Relapse	No	30	196			
	Yes	43	153	1.84(1.10,3.06)	2.21(1.25,3.91)	0.007
Depression	No	67	279	1.00	1.00	
	Yes	6	70	2.80(1.17,6.72)	1.49(0.58,3.83)	
Quality of life	Good	60	157	1.00	1.00	
	Poor	13	192	5.64(2.99,10.66)	5.57(2.79,11.09)	0.000

## Discussion

This study found that magnitude of General sexual dysfunction, and sexual dysfunction across male and female patients with Schizophrenia is extremely high and needs immediate intervention. The prevalence of general sexual dysfunction among patients with Schizophrenia in this study is supported by the comparative study conducted in Egypt on paranoid and non-paranoid schizophrenic patients in which Prevalence of general sexual dysfunction was 80% in patients with paranoid schizophrenia and 86.7% in patients with non-paranoid Schizophrenia [7]. The magnitude of general sexual dysfunction in this study is very high when compared to the study conducted in Britain and Iran which was 45 and 31.1% respectively [12, 13]. The reason for this significant difference may be explained by socio-cultural difference and difference in measurement instrument to assess sexual dysfunction. In case of the study conducted in Britain, they used Sexual Functioning Questionnaire (SFQ) to assess sexual dysfunction, and the study conducted in Iran used Arizona Sexual Experience Scale (ASEX) [12, 13].

Prevalence of sexual dysfunction among male Schizophrenic patients in our study which is 84.5% in lines with the study conducted by Macdonald in which the magnitude of sexual dysfunction in male Schizophrenic patients is 82% [14]. Magnitude of sexual dysfunction among female schizophrenic patients in this study which is 78.6% is lower than that of Macdonald in which prevalence of sexual dysfunction among female schizophrenic patients is 92%, and is higher than that of USA and Turkey in which prevalence of female sexual dysfunction is 59 and 68% respectively [14–16]. The reason for the discrepancy is probably due to difference in culture and living style which may differ in different countries and population. Difference in measurement tool to assess sexual dysfunction in this particular population is also possible reason for observed difference. In case of USA they used the Global Impression of Sexual Function (GISF) and in Turkey they used Arizona Sexual Experience Scale (ASEX) to assess sexual dysfunction [15, 16]. Another possible reason for the difference might be criterion used to include the participants in the study. The study conducted in USA included patients who were on conventional antipsychotics and Resperidone where as in our study all schizophrenic patients on any antipsychotic medication were included in the study [16]. Sample size difference across the studies is also possible reason for the discrepancy. Among the factors hypothesized to be contributing factors to sexual dysfunction, unmarried participants were four times more likely to develop sexual dysfunction compared to married participants and Divorced and widowed participants were three times more likely to develop sexual dysfunction compared to married participants. This finding is supported by the study conducted in Nigeria [17]. The possible reason for the association is that the infrequent sexual activity in single and divorced/widowed individuals probably decreases frequency of sexual desire. Having history of relapse exposes two times more to sexual dysfunction compared to the patients without history of relapse. This may be explained by, the more the relapse is frequent the more the illness becomes deteriorated with predomination of negative symptoms of Schizophrenia which potentially affect sexual performance. The need for higher doses of antipsychotics in case of frequent relapse is also another possible reason for this significant association. In this study sexual dysfunction is found to be highly associated with poor quality of life. This result is supported by the study conducted by Olfson and Kandrakonda S [18, 19]. This may be due to the fact that an inproper sexual functioning may affect maintaining a satisfying intimate relationship which is the major component of Quality of life.

## Conclusion

Prevalence of Sexual dysfunction is found to be high among patients with Schizophrenia and it needs special attention. The current prevalence of general sexual dysfunction among Schizophrenic patients in our study is high. Regarding sexual dysfunction across sex, male and female sexual dysfunction was also high. Among the hypothesized factors to be risk factors for sexual dysfunction, marital status (single, divorced, widowed), history of relapse and poor quality of life were significantly associated with sexual dysfunction.

## Recommendation

To Amanuel Mental Specialized Hospital.

All Psychiatrists and mental health specialists have to be conscious to sexual dysfunction which is highly prevalent among the patients they are treating, and all patients who are on follow up at this hospital for the case of Schizophrenia should be screened for sexual dysfunction. The overall treatment and care delivered by the hospital should focus on improving quality of life by diagnosing and managing sexual dysfunction properly, rather than focusing only on decreasing the symptom of the illness. Special consideration should be given to a patients with history of relapse, single, widowed and divorced.

## Abbreviations

AMSH: Amanuel Mental Specialized Hospital
CSFQ: Changes in Sexual Functioning Questionnaires
PHQ-9: Patients Health Questionnaire nine
SD: Sexual Dysfunction
SMI: Severe Mental Illness
SPSS: Statistical Package of Social Science
USA: United States of America
WHOQOL: World Health Organization Quality of Life

## References

[1] Zemishlany Z, Weizman A. The impact of mental illness on sexual dysfunction. InSexual Dysfunction 2008 Apr 8 (Vol. 29, pp. 89–106). Karger Publishers. 10.1159/000126626 18391559

[2] Baggaley M (2008). Sexual dysfunction in schizophrenia: focus on recent evidence. Hum Psychopharmacol Clin Exp.

[3] Kelly DL, Conley RR (2004). Sexuality and schizophrenia: a review. Schizophr Bull.

[4] Hanssens L, L'Italien G, Loze JY, Marcus RN, Pans M, Kerselaers W (2008). The effect of antipsychotic medication on sexual function and serum prolactin levels in community-treated schizophrenic patients: results from the schizophrenia trial of aripiprazole (STAR) study (NCT00237913). BMC psychiatry.

[5] Kikuchi T, Iwamoto K, Sasada K, Aleksic B, Yoshida K, Ozaki N (2012). Sexual dysfunction and hyperprolactinemia in Japanese schizophrenic patients taking antipsychotics. Prog Neuro-Psychopharmacol Biol Psychiatry.

[6] Hashem A.H.*,* Abd El-Gawad T.*,* Ezzat M.*,* Assal A.*,* Goueily T. *and* El Rakhawy M. A comparative study of sexual function in paranoid versus non-paranoid schizophrenic patients and its relation to serum prolactin Level.Current psychiatry. Vol 13. No. 2. July 2006.

[7] Maria Paz Garcia-Portilla, MD, PhD et al. Psychometric properties of the Spanish version of the changes in sexual functioning questionnaire short-form (CSFQ-14) in patients with severe mental disorders. International Society for Sexual Medicine J Sex Med 2011;8:1371–1382.**.** 10.1111/j.1743-6109.2010.02043.x 20946156

[8] Liu-Seifert H, Kinon BJ, Tennant CJ, Sniadecki J, Volavka J (2009). Sexual dysfunction in patients with schizophrenia treated with conventional antipsychotics or risperidone. Neuropsychiatr Dis Treat.

[9] Oyekanmi AK, Adelufosi AO, Abayomi O, Adebowale TO (2012). Demographic and clinical correlates of sexual dysfunction among Nigerian male outpatients on conventional antipsychotic medications. BMC research notes.

[10] Olfson M, Uttaro T, Carson WH, Tafesse E (2005). Male sexual dysfunction and quality of life in schizophrenia. J Clin psychiatry.

[11] Kandrakonda S, Jally MR, Kesava Reddy SR, Miryala G (2014). Prevalence of sexual dysfunction in patients with mental illness receiving psychotropic medication. AP J Psychol Med.

[12] CLAYTON ELMaAH. Reliability and construct validity of the changes in sexual functioning questionnaire short-form (CSFQ-14):. Journal of Sex & Marital Therapy,. 2006;32:43–52. Departments of Psychiatric Medicine & Health Evaluation Sciences, University of Virginia, Charlottesville, Virginia, USA. 10.1080/00926230500232909 16234225

[13] WHO. WHOQOL, user manual, division of mental health and prevention of substance abuse. Geneva, Switzerland.. 1998.

[14] Gelaye B, et al. Validity of the patient health Questionnaire-9 for depression screening and diagnosis in East Africa. Psychiatry Res. 2013 December 15;210(2) 10.1016/j.psychres.2013.07.015. 10.1016/j.psychres.2013.07.015 PMC3818385 23972787

[15] Morisky E, Ang A, Wood M (2008). Predictive validity of a medication adherence measure in an outpatient setting. J Clin Hypertens (Greenwich).

[16] Smith S, O'KEANE VE, Murray R (2002). Sexual dysfunction in patients taking conventional antipsychotic medication. Br J Psychiatry.

[17] Ahmadzadeh G, Shahin A. Sexual dysfunctions in the patients hospitalized in psychiatric wards compared to other specialized wards in Isfahan, Iran, in 2012. Advanced biomedical research. 2015;4–225. 10.4103/2277-9175.166648 PMC4638051 26623400

[18] Macdonald S, Halliday J, MacEwan T, Sharkey V, Farrington S, Wall S, McCreadie RG (2003). Nithsdale schizophrenia surveys 24: sexual dysfunction. Br J Psychiatry.

[19] Hocaoglu C, Celik FH, Kandemir G, Guveli H, Bahceci B (2014). Sexual dysfunction in outpatients with schizophrenia in Turkey: a cross-sectional study. Shanghai Arch Psychiatry.

